# Right to health: (in) congruence between the legal framework and the health system

**DOI:** 10.1590/1518-8345.0995.2679

**Published:** 2016-03-28

**Authors:** Fernando Mitano, Carla Aparecida Arena Ventura, Mônica Cristina Ribeiro Alexandre d'Auria de Lima, Juvenal Bazilashe Balegamire, Pedro Fredemir Palha

**Affiliations:** 1 Supported by Fundação de Apoio à Universidade Estadual do Paraná, Brazil.; 2 Doctoral student, Escola de Enfermagem de Ribeirão Preto, Universidade de São Paulo, PAHO/WHO Collaborating Centre for Nursing Research Development, Ribeirão Preto, SP, Brazil. Assistant Professor, Universidade Estadual do Paraná, Paranavaí, PR, Brazil; 3 Undergraduate student in Nursing, Universidade Estadual do Paraná, Paranavaí, PR, Brazil. Scholarship holder from Fundação Araucária, Brazil; 4 PhD, Professor, Universidade Estadual de Maringá, Maringá, PR, Brazil

**Keywords:** Right to Health, Health Systems, Health Services Coverage, Health Services Accessibility

## Abstract

**Objective:**

to discuss the right to health, incorporation into the legal instruments and the deployment in practice in the National Health System in Mozambique.

**Method:**

this is a documentary analysis of a qualitative nature, which after thorough and interpretative reading of the legal instruments and articles that deal with the right to health, access and universal coverage, resulted in the construction of three empirical categories: instruments of humans rights and their interrelationship with the development of the right to health; the national health system in Mozambique; gaps between theory and practice in the consolidation of the right to health in the country.

**Results:**

Mozambique ratified several international and regional legal instruments (of Africa) that deal with the right to health and which are ensured in its Constitution. However, their incorporation into the National Health Service have been limited because it can not provide access and universal coverage to health services in an equitable manner throughout its territorial extension and in the different levels of care.

**Conclusions:**

the implementation of the right to health is complex and will require mobilization of the state and political financial, educational, technological, housing, sanitation and management actions, as well as ensuring access to health, and universal coverage.

## Introduction

The right to health is part of the context of social rights, one of the most difficult rights to protect, especially when civil and political rights are considered^(^ 1 ^)^, as it implies effective and proactive actions of the states, in order to attain it, by proposition and assurance of policies and programs in the health field. Nevertheless, good health is desired by every human being as a condition for exercising the rule of law. Talking about the right to health implies reference to a set of legal norms that establish the rights and obligations of the state, collectively and also individually, regulating and monitoring the relationships between them^(^ 1 ^)^. It also refers to access and universal coverage of health services, in quality and quantity for the whole population.

Universal health service coverage should offer high quality services and access, also prioritizing promotion, prevention, treatment, rehabilitation and palliative care^(^ 2 ^-^ 4 ^)^. In this context, the health systems and their governments, to guarantee this coverage, should act on the social and environmental determinants of health^(^ 3 ^-^ 5 ^)^, pointing out that the performance of these factors should be a task of all state sectors.

In many countries, especially developing countries such as Mozambique, there are several obstacles to accessing healthcare services, which are an obstacle to universal coverage. Among these are: lack of sanitary infrastructure, insufficient human resources, lack of equipment, higher demand, difficulty of paying users for the services performed, professional attitudes and characteristics of health services^(^ 5 ^)^. All of these barriers contribute to the deprivation of the right to health of populations.

The independence of Mozambique from Portuguese domination in 1975 did not necessarily mean the full exercise of the right to health for most of the population. Rather, the great challenge is exactly to improve the high rate of chronic malnutrition, especially in children, high food insecurity, low levels of education of women, poor access to safe drinking water, inadequate levels of availability to basic sanitation and access to high quality health services, and the equitable distribution of health care professionals in urban, peripheral and rural areas where health needs are higher^(^ 3 ^)^.

These are some of the challenges that reveal the difficulties of the State in operationalizing coverage targets and universal access to health, caused also by the lack of funds for the health system, deficient information systems, negative relationships between health professionals and the people, and late referral for more specialized care of patients with certain conditions from the clinics of lower levels of care ^(^ 3 ^-^ 5 ^)^. Access to health services becomes effective when accompanied by universal coverage, understood as the development of health financing systems by the State that allow people to use services and health actions without financial burden, such as direct disbursement to obtaining needed health care^(^ 3 ^,^ 6 ^)^. This requires that services are provided equitably, regardless of geographic location, cultural values, beliefs, religion and ethnicity. Lack of access and universal coverage constitutes a barrier for realization of the right to health in Mozambique.

Aiming at deepening this subject, a literature review was performed in the databases of the Latin American and Caribbean Literature in Health Sciences - (LILACS), PubMed and *Scopus* , however, no article on Mozambique was found. For academic contribution to this discussion, legislation about the right to health was used, elaborating some questions that will guide this reflective study: what international and regional instruments ratified the right to health in Mozambique? What are the legal instruments in Mozambique that include the right to health? With what theoretical, legal and social frameworks can the gap between theory and practice on the right to health in Mozambique be understood? To answer these questions, the article presents as its objective the discussion on the right to health, incorporation into legal instruments and the deployment in practice in the National Health System in Mozambique.

## Method

This was a documentary analysis of a qualitative approach, which followed the guidelines of Souza, Kamorski and Luis^(^ 7 ^)^ and Viswambharan and Priya^(^ 8 ^)^, taking documentary analysis as an analytical process, from documentary sources, which enables the identification, verification and examination of documents, expanding and questioning the background. The use of document analysis extends the knowledge, whose understanding requires a contextual historical approach, allowing the comprehension of the processes of evolution of practices, behaviors, trends and application of certain measures^(^ 6 ^)^.

For this study, all official legislation of Mozambique on the right to health published in the Bulletin of the Republic of Mozambique between 1975 and 2010, were used, in addition to the Constitution. The African Charter on Human and Peoples' Rights and the International Covenant on Economic, Social and Cultural Rights were also consulted. 

After exhaustive reading of the documents, three empirical categories were established: "instruments of humans rights and their interrelationship with the development of the right to health"; "the national health system in Mozambique"; and "gaps between theory and practice in the consolidation of the right to health in the country."

## Results

**Figure 1-:**
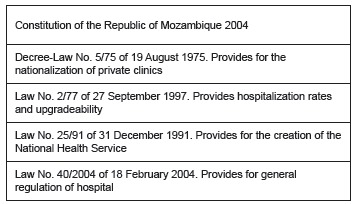
Legislative instruments analyzed that provide for the right to health in Mozambique

Five national legislative documents were analyzed: the Constitution of the Republic of Mozambique, 2004; a decree-law; and three laws. Figure 1 shows these legal documents.

Then, the three empirical categories were analyzed, mobilizing the bibliography that shows the situation of the existence of the right to health or lack thereof, coverage and access to health services.

## Instruments of humans rights and their interrelationship with the development of the right to health in Mozambique

**Figure 2:**
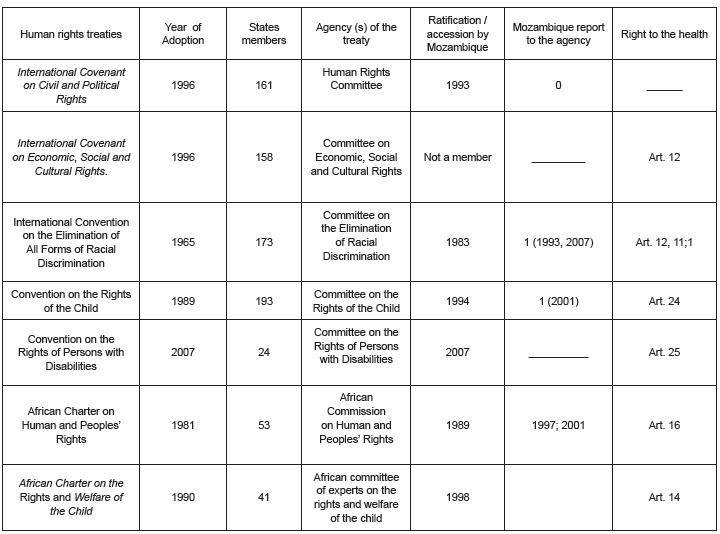
- International instruments on human rights and the link or lack thereof of these instruments from Mozambique

Source: Adapted from Mozambique - WHO - General Fact Sheet on the right to health, available from: http://www.ohchr.org (9).

When the nation, a member of the World Health Organization and the African Union, approved instruments affirming the right to health, Mozambique was still a Portuguese colony, without the legal structure to include this prerogative into the rule of law. But, with its independence in 1975, Mozambique acceded to several international and regional human rights instruments that directly or indirectly addressed the guarantee of the right to health. For a better illustration, Figure 2 shows some human rights instruments, with some content related to health and the position of Mozambique.

Mozambique participates as a member state of several international and regional treaties relevant to the implementation of the right to health, although it has not ratified the International Covenant on Economic, Social and Cultural Rights. 

In the domestic context, Mozambique has important tools that are able to guarantee the right to health, especially the 2004 Constitution of the Republic^(^ 10 ^)^, which explicitly states the responsibility of the State to provide access to health services for all Mozambicans in Article 49, "all citizens have the right to medical and health care, according to the law and the duty to promote and protect public health". Although the previously mentioned article refers to the guarantee to health, the Constitution does not clearly explain the country's obligation to provide equitable health services to all.

In addition to the constitutionality of guaranteeing the right to health for the population, Mozambique, through its Constitution, emphasizes the importance of social participation in health, especially on the promotion and protection of public health, with the involvement of Community bases^(^ 2 ^)^, although, it does not indicate which instruments, or constitutional- or intra-constitutional instruments the state will use for social participation.

Regarding the State's responsibility, the African Charter on Human and Peoples' Rights, ratified by the government of Mozambique, mentions in Article 16 that "everyone has the right to enjoy the best health that he is able to achieve" and reinforces also that "the member states of this charter are committed to taking the necessary measures to protect the health of their populations and to assure medical care in case of illness"^(^ 11 ^)^. The Charter implies the mission of any member state to the commitment to ensure the health of its population, while it does not assure the guarantee as a responsibility or duty of the state, however, it reinforces individual freedom to enjoy the best health that one is capable of obtaining.

Mozambique still faces major challenges to the equitable offering of the status of right to health, not only in improving health services, but in ensuring effective and broad actions, which act positively on the social determinants that affect the right to health. In this sense, the actions for the fight against food insecurity, public safety, supply and access to clean water, sanitary measures in private and public environments, access to basic and higher education, environmental sanitation, poverty reduction policies, and the consumption of alcohol, drugs and tobacco, which are social determinants that limit access and universal coverage for health services^(^ 3 ^,^ 5 ^)^. 

## The National Health System in Mozambique

Upon the independence of Mozambique, the Council of Ministers, through Decree-Law N. 5/75 of 19 August 1975^(^ 12 ^)^, nationalized the private clinics that existed during the colonial period, in order to maximize resources by offering greater access to health care services that provided indiscriminate assistance to Mozambican citizens. The Decree, in Article 1, states that "all activities relating to the prevention and treatment of disease as well as the technical preparation of the staff team must be exclusively performed by the State". Therefore, all activities of primary, secondary and tertiary care became the sole and exclusive responsibility of the State, which became the sole provider and promoter of health services for the entire country, thereby assuming control of Portuguese hospitals and private clinics^(^ 12 ^)^. This context is derived from a political position of a government with socialist characteristics, which emphasizes access to health and pharmaceutical care for the Mozambican population through the principles of equality and equity.

In 1977 this situation changed as a result of Law N. 2/77^(^ 13 ^)^, which emphasizes in Article 1 the gratuity of prophylactic health actions. In this regard, the Ministry of Health (MoH) adopted a payment criterion with a fixed value for medical and pharmaceutical appointments in the 11 provinces of the country, considered affordable prices for everyone.

The adoption of participation through payments used to limit access of the population to health services, as the country at that time had a high unemployment rate (30%) and a high illiteracy rate (68%)^(^ 4 ^)^, factors that occasioned misinformation and the impossibility of having what was established by the MoH. Associated with these facts, most of the population lived in rural areas where health services were less available, further aggravating the right to access to health.

In 1991 new changes are perceived through the creation of the National Health System (NHS), Law N. 25/91^(^ 14 ^)^in light of the Republic Constitution of 1990, which established the right to medical and health care for all citizens through the MoH. Its goal was health promotion, disease prevention, care and rehabilitation, associated with training of human resources and research for its continued development. Thus, the NHS established a health organization at different levels of care: primary level - consists of health centers and units, each comprising its respective areas of health; secondary level - consists of district general and rural hospitals; tertiary level - consists of provincial hospitals; and quaternary level - consists of central and specialized hospitals. The institutions that are part of the NHS under the MoH management are the hygiene centers, vocational training institutions, specialized laboratories and research institutions.

The NHS centralizes financial resources and organizes the technical instructions of the MoH hierarchically by the provincial guidelines. Thus, the Central Government determines the percentage of the State's general budget for the MoH, which varies from year to year, and the State distributes it to the provinces. This centralization means that financial resources are channeled mostly to health facilities in the tertiary and quaternary levels, to the detriment of health centers, which are mostly in rural and peripheral areas of the cities, thereby reducing access of the population to health facilities, which interferes with the full right of access to health services for the population living far from urban centers.

Another factor that must be considered is that the MoH depends on donations of international organizations and partner countries, especially Western, to perform its actions. These resources do not always meet the needs for coverage and access to health services for the population, not only because they are scarce, but also because they are not geographically allocated equitably, giving privileges to high-complexity hospitals, directly interfering with the population's right to health^(^ 2 ^-^ 6 ^)^.

Resuming the discussions on nationalization, in 1991 during the advent of the democratization, the NHS admitted its inability to be the only provider of health services, resuming the activities of private health care, which had been nationalized after the independence of the State. On the other hand, in 2004, the State officially recognized the need for activities of practitioners of traditional medicine by approving the traditional medicine policy and its implementation strategy, whose main objective was its integration to the NHS to guarantee access to primary health care and quality for the entire Mozambican population^(^ 15 ^)^.

This recognition represents advances that the State has been providing since its independence for the consolidation of the traditional medicine area, with the creation of the Traditional Medicine Research Office in 1977, and the creation of the Association of Traditional Physicians of Mozambique^(^ 15 ^)^. This recognition does not seem to be necessarily valuing this health practice and the importance of traditional physicians, but rather highlights a limitation of the NHS in offering universal access to health services and coverage to the entire population, since in this period the NHS covered only 40% of services, and the rest were covered by traditional health practitioners and private health care. It should be noted that even given every effort by the State, the NHS had covered only about 50% by 2013^(^ 4 ^,^ 8 ^)^.

The fact that traditional medicine has been recognized as an NHS participant undoubtedly adds value to the right to health and respect for local culture, to the extent that people can consult traditional physicians freely. However, there is still no effective integration of these health practices within the NHS, thereby not ensuring the regulation of the MoH on the supply of these services and systematized protocols with referral and counter-referral that enable the coordination of care in a more articulate manner, making the guarantee to the right to health even more difficult. It is important to note that traditional medicine covers 100% of all of the Mozambican population, namely, even people who have access to the NHS also access the traditional medicine system.

## Gaps between theory and practice in the consolidation of the right to health in the country

Although the right to health depends on specific legislation to ensure rights and duties, it requires mutual efforts to function. In this context, the importance of availability is emphasized, which involves the construction of infrastructure and the existence of health care services in a sufficient amount for the whole population; accessibility, which involves health care services that are available to the entire population without discrimination; physical accessibility, in which health facilities are available to all, including the reduction of distances and means of transportation available to meet the needs of users, greater attention to the most vulnerable people in the communities; and accessibility, which implies the recognition of the difficulties of access to work, and greater equity in payments, which should be as accessible as possible for all when they exist; the population's access to information without discrimination and the duty of health professionals to respect medical ethics and recognize the cultural issues in the contexts in which they work can be an obstacle in care^(^ 3 ^-^ 6 ^)^.

Finally, in order to ensure this right, there must be quality services, equipment and human resources^(^ 3 ^)^. In Mozambique, where most of the population is unemployed, and although the payment amounts are "symbolic", they interfere with access and universal health coverage of services, in costs of medical consultation, medicines, and mainly in transportation for those who live in locations that are far from health facilities.

The right to health has been established as a regulated and progressive implementation right, as the lines or principles for the fulfillment of State programs are conceived^(^ 1 ^)^. In this context, the States make an effort to implement the right to health, according to its financial conditions, as stated by Pact 1966: "each Member State in the present Pact is committed to adopting measures, (...) especially in the economic plans and technical contexts, to the maximum of its available resources in order to progressively achieve (...), the full realization of the rights recognized in the present Pact, including (...) the adoption of legislative measures"^(^ 16 ^)^. Most importantly, in this process of right to health, is the recognition that the State has an immediate duty to fulfill essential obligations, having as responsibilities to regulate, design and implement policies and mobilize financial resources.

For the right to health to be effectively implemented for the population, empowerment would be required, including universal access to education, health, employment, promotion of equality of gender within ethnic groups, and the institutional ability to respond^(^ 1 ^-^ 2 ^,^ 6 ^,^ 11 ^)^. A community-based intervention would also be important to guarantee access to a wide variety of promotional, preventive, curative and rehabilitative health services^(^ 3 ^-^ 4 ^)^.

But what happens in Mozambique? In order to have the right to health, financial resources for the various levels of the NHS and other health subsystems are essential. among other factors; political decentralization in the implementation and coordination, so that decisions are made at other levels (central, provincial and district); and medical technology, which involves a set of tools and processes that effectively respond to health care needs^(^ 17 ^)^.

The challenges presented, and one of the aspects to be considered, relate to the financing arranged by the Mozambican state, one of the lowest in the Southern African region, with an external dependence for about 66% of their spending. Mozambique spends approximately 39 dollars per capita on health, representing much less compared to the regional average^(^ 4 ^)^. Although the gross domestic product (GDP) has been growing in recent years, around 7% per year, the budget allocated to the NHS state is 6.6%, below the recommended 15%in the Africa region ^(^ 4 ^)^.

This decentralization and transparency are not well defined by the MoH, since there is no visibility on accountability for the population as a whole, although there is a set of policies, strategies, laws and regulations^(^ 3 ^,^ 8 ^,^ 12 ^)^. The country lacks quantity and quality medical technology, materials and processes^(^ 3 ^-^ 4 ^,^ 15 ^)^; therefore it does not have sufficient infrastructure to fully meet the access to diagnostic tools that are able to respond safely to the laboratory diagnosis of different diseases that affect the population. Although the coverage of health services has improved, it is still low to meet all the health needs of the population^(^ 4 ^,^ 13 ^,^ 17 ^)^.

One cannot deny the improvements in some health areas, such as the increase of births attended by health professionals, set at 54.3%; reducing poverty by almost 50%; and reducing neonatal mortality from 50% to 30.4%^(^ 8 ^,^ 4 ^,^ 13 ^)^; but the state of the right to health is still far from being effected within the whole population. Other factors associated with lack of coverage of health services in countries such as Mozambique are related to social inequalities, government political systems^(^ 3 ^-^ 6 ^)^ and, in the case of the African countries, political and economic instability is added. For countries with these characteristics, the universal service coverage requires reforming systems, promoting the principles of equity, solidarity and collective action to overcome inequalities^(^ 3 ^-^ 5 ^)^, since the absence of all this cannot guarantee universal access and coverage and consequently, the right to health.

## Conclusion

The theme of the right to health is complex, as permeated by various spheres, including health, law, financial policies, education, technology, housing, sanitation and management.

In Mozambique, the State's actions, represented by the NHS, show the gap between the recommended policies and health practices, with an incipient focus on equity and quality, although there is an increased demand for health services systematically.

The difficulties of access and universal coverage, and hence the establishment of the right to health, is a reflection of: underfunding in health; difficulties decentralizing care processes and medical technologies; minimal coverage of NHS; lack of qualified professionals and a pricing policy to maintain these professionals; difficulty with work conditions and processes in health care; few activities in the interdisciplinary and teamwork fields; and systematic practice of professionals, such as nurses.

For the people to have the right to health, in addition to the actions implemented by the government, there is also the need for participation of the population, by using social control mechanisms, requiring greater access and coverage of health services by the State.

For the general population of Mozambique, traditional medicine practices can consist of an expansion of universal access and coverage of health services that are contracted, supervised and regulated by the State.
